# Advances in the prevention of respiratory syncytial virus infection in children

**DOI:** 10.36416/1806-3756/e20250469

**Published:** 2026-03-05

**Authors:** Júlia Giffoni Krey, Laura Manfroi, Francine Bester Damian, Leonardo Araújo Pinto, Marcelo Comerlato Scotta

**Affiliations:** 1. Grupo de Pesquisa em Epidemiologia e Genética das Doenças Respiratórias da Infância, Escola de Medicina, Pontifícia Universidade Católica do Rio Grande do Sul - PUCRS - Porto Alegre (RS) Brasil.; 2. Programa de Pós-Graduação em Medicina - Pediatria, Escola de Medicina, Pontifícia Universidade Católica do Rio Grande do Sul - PUCRS - Porto Alegre (RS) Brasil.

## INTRODUCTION

Respiratory syncytial virus (RSV) is a leading cause of hospitalization in infants. Recent advances have transformed prevention strategies, with maternal vaccination and long-acting monoclonal antibodies showing high efficacy. The present paper reviews the impact of these new prevention strategies. The bivalent prefusion F protein-based RSV vaccine (RSVpreF, Abrysvo; Pfizer) given during pregnancy has been shown to significantly reduce RSV-related illness in infants.^1^ ^,^ ^2^ Moreover, new monoclonal antibodies, including nirsevimab (Beyfortus; AstraZeneca/Sanofi) and clesrovimab (Enflonsia; Merck), have also proven highly effective in preventing severe disease.^3^ Together, these interventions represent major progress toward universal prevention of RSV-related illness in early childhood.

## MATERNAL VACCINE FOR RSV

The implementation of maternal vaccination has been one of the main modern strategies for preventing RSV infection in infants. This approach results in the transplacental transfer of maternal antibodies at increased levels, aiming to provide passive immunity to children immediately after birth and during the first months of life, particularly in the first six months, which is the highest-risk period for severe RSV disease. The pivotal study of the RSVpreF vaccine included pregnant individuals vaccinated at 24-36 weeks of gestation.^1^ ^,^ ^2^ In the United Kingdom, the recommendation was to vaccinate from 28 weeks onward. Previously, the Brazilian Society of Immunizations, from the point of view of individual health, suggested vaccination between 32 and 36 weeks of gestation.

Randomized controlled trials have demonstrated that maternal RSV vaccination significantly reduces the risk of severe RSV-associated lower respiratory tract infection and RSV-related hospitalization in children by up to 80% during the first three months. The vaccine is recommended as a single dose during pregnancy, and the use of simultaneous maternal vaccination and monoclonal antibodies for infants is still controversial as a routine practice, except in rare circumstances. The most common side effects are mild and include injection site pain, headache, myalgia, and nausea. Although data from the subgroup of low- and middle-income countries in the pivotal study showed an association with preterm birth, real-world studies indicate that maternal RSV vaccination does not increase that risk or the risk of congenital abnormalities, intrauterine growth restriction, stillbirth, maternal death, or infant death.^3^ ^-^ ^5^

## MONOCLONAL ANTIBODIES FOR RSV

Nirsevimab is a long-acting monoclonal antibody that represents a significant advance in the prevention of RSV-related illness. Pivotal studies in premature and term babies have shown an efficacy around 75-80% for RSV-related hospitalizations. Furthermore, real-world data from many countries reinforce these results, showing similar numbers for effectiveness.^4^ According to the Advisory Committee on Immunization Practices (ACIP) recommendations, this antibody should be administered as a single dose to all infants under eight months of age who are born during or entering their first RSV season.^1^ In addition, a single dose is also recommended for children aged eight to nineteen months who are at increased risk for severe RSV disease-such as immunocompromised children or those with certain chronic conditions-and who are entering their second RSV season.^3^ This approach aims to provide universal protection, replacing the previous strategy that focused solely on high-risk groups.

Clesrovimab is the second long-acting monoclonal antibody licensed for the prevention of RSV and serves as an alternative to nirsevimab.^6^ Its indication is the same: to protect infants under eight months of age against severe lower respiratory tract infection associated with RSV through a single dose administered during their first RSV season.^2^. Its introduction reinforces the ACIP strategy of ensuring that all infants have access to prophylaxis, with the choice between nirsevimab and clesrovimab depending on product availability, clinical preference, and shared decision-making between parents and healthcare providers.^2^ ^,^ ^3^

The short-acting monoclonal antibody palivizumab (Synagis; Medimmune) has been used for many years for the prevention of RSV-related illness, but with a very different approach. Unlike the new single-dose antibodies, the use of palivizumab requires monthly doses during the RSV season. This treatment was specifically reserved for children considered high-risk, with underlying medical conditions such as certain congenital heart diseases or bronchopulmonary dysplasia. Furthermore, there has been a paradigm shift, with the long-acting antibodies (nirsevimab and clesrovimab) becoming the preferred options and palivizumab being mentioned in the historical context for high-risk groups.^2^ ^,^ ^3^

## CONCLUSIONS

The maternal vaccine and long-acting monoclonal antibodies are both highly effective and strongly recommended in the current guidelines. The choice between these methods may depend on product availability, the relationship between time of birth and RSV season, maternal health status and patient preference. Maternal vaccination provides polyclonal antibody responses, potentially offering broader protection against viral variants, whereas monoclonal antibodies ensure direct delivery of protective antibodies to the child. In summary, these strategies represent major advances in the prevention of RSV-related illness, with robust evidence for efficacy and safety, and early data demonstrate significant reductions in the incidence of severe RSV-related disease in young infants (Chart 1).

**Chart 1:** Comparison between emerging approaches in the prevention of respiratory syncytial virus-related illness.

Parameter	Maternal RSVpreF vaccine	Nirsevimab
Target population	Pregnant women at 24-36 weeks of gestation with singleton, uncomplicated pregnancies	All preterm and term newborns/infants ≤ 12 months of age, or entering their first RSV season; infants with comorbidities entering their second season
Type of immunization	Active immunization-maternal vaccination induces antibody production	Passive immunization-administration of a ready-made monoclonal antibody
Infants covered by the indication	All infants born to vaccinated mothers, regardless of prematurity (if maternal vaccine given ≥14 days before delivery)	All preterm and term newborns/infants ≤ 12 months of age, or entering their first RSV season; infants with comorbidities entering their second season
Timing of administration	Single intramuscular dose (120 µg) administered to the mother during pregnancy (24-36 weeks)	Single intramuscular dose administered directly to the infant before the start of the RSV season
Seasonal consideration	Administered during pregnancy before the expected RSV season to ensure infant antibody protection at birth	Administered shortly before or at the beginning of the RSV season
Primary prevention goal	Prevent RSV-associated lower respiratory tract infection and hospitalization during the first RSV season	Prevent RSV-associated lower respiratory tract infection and hospitalization during the first RSV season
Duration of protection	Protection for approximately 6 months after birth	Protection lasting for at least 5 months post-injection
Advantages	Protects infants from birth, avoiding injection in the newborn	Provides consistent protection regardless of maternal factors (e.g., timing, antibody transfer)
Limitations	Effectiveness depends on the timing of vaccination and adequate placental transfer; may not protect premature infants born soon after vaccination	Requires direct administration to the infant; may not cover those born after the RSV season

RSV: respiratory syncytial virus; and RSVpreF: (bivalent) prefusion F protein-based RSV vaccine.
